# Gender-Based Impact of Epidermal Growth Factor Receptor Mutation in Patients With Nonsmall Cell Lung Cancer and Previous Tuberculosis: Erratum

**DOI:** 10.1097/MD.0000000000008578

**Published:** 2017-11-03

In the article, “Gender-Based Impact of Epidermal Growth Factor Receptor Mutation in Patients With Nonsmall Cell Lung Cancer and Previous Tuberculosis”,^[1]^ which appeared in Volume 94, Issue 4 of *Medicine*, Dr. Chih-Hsin Lee's affiliation should have appeared as Division of Pulmonary Medicine, Department of Internal Medicine, Wanfang Hospital and Division of Pulmonary Medicine, Department of Internal Medicine, School of Medicine, College of Medicine, Taipei Medical University, Taipei, Taiwan.
